# pcaReduce: hierarchical clustering of single cell transcriptional profiles

**DOI:** 10.1186/s12859-016-0984-y

**Published:** 2016-03-22

**Authors:** Justina žurauskienė, Christopher Yau

**Affiliations:** Wellcome Trust Centre for Human Genetics, University of Oxford, Roosevelt Drive, Oxford, OX3 7BN UK; Department of Statistics, University of Oxford, 1 S. Parks Rd, Oxford, OX1 3TG UK

**Keywords:** Single cell RNA-Seq, Hierarchical clustering, Gene expression

## Abstract

**Background:**

Advances in single cell genomics provide a way of routinely generating transcriptomics data at the single cell level. A frequent requirement of single cell expression analysis is the identification of novel patterns of heterogeneity across single cells that might explain complex cellular states or tissue composition. To date, classical statistical analysis tools have being routinely applied, but there is considerable scope for the development of novel statistical approaches that are better adapted to the challenges of inferring cellular hierarchies.

**Results:**

We have developed a novel agglomerative clustering method that we call *pcaReduce* to generate a cell state hierarchy where each cluster branch is associated with a principal component of variation that can be used to differentiate two cell states. Using two real single cell datasets, we compared our approach to other commonly used statistical techniques, such as *K*-means and hierarchical clustering. We found that *pcaReduce* was able to give more consistent clustering structures when compared to broad and detailed cell type labels.

**Conclusions:**

Our novel integration of principal components analysis and hierarchical clustering establishes a connection between the representation of the expression data and the number of cell types that can be discovered. In doing so we found that *pcaReduce* performs better than either technique in isolation in terms of characterising putative cell states. Our methodology is complimentary to other single cell clustering techniques and adds to a growing palette of single cell bioinformatics tools for profiling heterogeneous cell populations.

**Electronic supplementary material:**

The online version of this article (doi:10.1186/s12859-016-0984-y) contains supplementary material, which is available to authorized users.

## Background

Recent advances in single cell RNA sequencing (scRNA-seq) technology has enabled the routine high-throughput collection of quantitative gene expression measurements across a range of tissue types and diversity of cellular states at the level of the single cell [1–6]. The application of single cell gene expression profiling has identified cell-to-cell expression variability in phenotypically and/or genetically identical cells that is masked in standard “population” gene expression studies where the transcriptomes of thousands to millions of cells are simultaneously measured and averaged. This expression variability is driven by stochastic gene expression mechanisms whose effects cannot be measured in the context of a population of cells but only through the microcosm of a single cell. Consequently, scRNA-seq has increasingly become the method of choice in discovering molecular underpinnings of complex and rare cell populations [7, 8], assessing tissue composition [9–11], studying various diseases [12] and cell development/lineage processes [13–16].

Our particular focus in this article is the utility of scRNA-seq data to enable the identification of functionally distinct sub-populations that each possesses a different pattern of gene expression activity [17, 18]. These sub-populations could indicate different *cell types* that exhibit relatively stable, static behaviour but also *cell states* representing intermediate stages in transient processes. Traditionally, cell types have been defined by the functional behaviour of certain cellular features, for example, CD14+ monocytes show CD14 expression, but with the availability of scRNA-seq the potential exists to develop a richer taxonomy of cell types by extending the molecular features used for characterisation to consider the whole transcriptome. The population of CD14 expressing monocytes might in fact be a collection of distinct cell subtypes each sharing a common CD14 expression signature but also possessing a unique expression pattern of their own.

Unbiased discovery of cell types from scRNA-seq data can be automated using unsupervised clustering algorithms. Given expression profiles for a collection of single cells, the objective of the algorithm is to partition the cells into a number of cell types such that each cell type has a significantly distinctive expression signature from the others. Single cell analytical software pipelines have been developed recently for single cell analysis that include procedures for unbiased cell type identification.

In RaceID [19], *K*-means clustering with gap statistics was used to identify six intestinal cell types, while rare cell types were identified by examining outliers that could not be explained by a background noise model. The BackSPIN method [9] uses a customized version of the SPIN algorithm [20]. When applying the BackSPIN method to 3005 mouse cortex and hippocampus cells, the algorithm identified 77 groups, stopping after 12 splits along the deepest branch. This was manually limited to 5 splits per branch and subsequent merging of the neuronal groups into three main groups (each containing four subgroups) and oligodendrocytes into one group (containing 3 subgroups) resulted in the nine main cell classes given in the study [9]. Seurat [13, 21] applies a two-dimensional t-SNE projection [22] to the most significant principal component scores (a process they refer to as “spectral t-SNE”) from a principal component analysis of the single cell expression matrix. Density clustering (DBSCAN) [23] is then used to identify cell type clusters in the two-dimensional space. Seurat was used to classify 44,808 Drop-seq single cell expression profiles into 39 retinal cell populations [21]. The SINCERA package [24] uses hierarchical clustering using centered Pearson’s correlation and average linkage as default settings for the similarity measurement and linkage method respectively. SNN-cliq [25] uses the concept of shared nearest neighbour (SNN) to define similarities between data points (cells) and achieves clustering by a graph theory-based algorithm. Finally, the SC3 approach [26] which uses spectral transformations, the *K*-means algorithm and complete-linkage to perform consensus clustering.

**Fig. 1:**
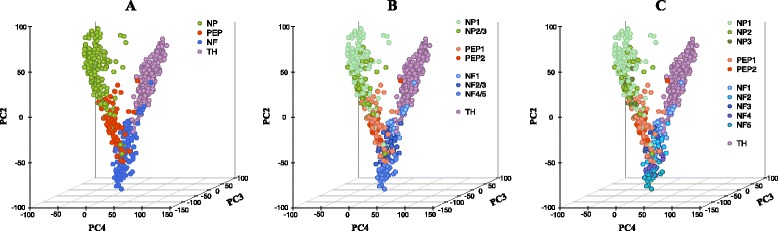
Cellular hierarchies. Three hierarchically related clustering structures for a single cell mouse neuronal dataset [27]. The data has been projected on to the first four principle directions, we report the three that allows best data visualisation; we used the given cellular labels to colour cells according to the **a** 4, **b** 8, and **c** 11 cell subtypes identified in the original study

A limitation of these methods is that they do not establish a connection between the *representation* of the data to the number and nature of the cell types that can be resolved. For example, Fig. 1 illustrates three clustering structures derived from a single cell study of mouse sensory neurons [27]. Four broad sensory neuronal cell types (NF, TH, PEP, NP) were identified by examining clusters of cells in the subspace spanned by the first few principal components (PC2-4 shown in Fig. 1a) and using expression of key (known) cell markers to label the clusters. Using information contained in additional principal components, the four major cell types could then be sub-divided into further distinct cell subtypes. The presence of these refined cell subtypes is clearly not obvious from a visual inspection of the data in the subspace spanned by PC2-4 (Fig. 1b,c).

We have developed an agglomerative clustering approach that integrates principal components analysis (PCA) and hierarchical clustering that we call *pcaReduce*. This method seeks to establish a connection between the reduced representations given by principal components analysis and the number of resolvable cell types (clusters). The approach is driven by the expectation that information pertaining to large, broad classes of cell types are likely be contained in low dimensional PC representations whilst refined cell type structure are only defined in high dimensional PC representations. Our proposed method is similar to the “iterative” principal component analysis approaches used to establish putative cell types in [27, 28]. However, the question we asked was whether we could recapitulate such results fully automatically in an unsupervised fashion without prior knowledge of cell type markers that was used in these studies. This question is important in study conditions where there maybe little or unreliable prior knowledge of cell types. We will test our methods against existing approaches but note that many existing approaches typically utilise different combinations of standard dimensionality reduction and clustering algorithms. Therefore, in our investigations, instead of using these packages directly, we will explore the utility of the constituents components (which might be shared between approaches).

## Methods

Let **X**_*n*×*d*_ denote a gene expression matrix, where *n* is the number of cells measured across *d* number of genes; i.e. each cell **x**_*i*_ = {*x*_*i*1_,…,*x*_*id*_} is a *d*-dimensional object. Further assume that **Y**_*n*×*q*_ denotes a score matrix, obtained after projecting data into first *q* principle directions, and *Y*_*i*_ denotes a subset of cells, *Y*_*i*_⊂**Y**.

Our clustering algorithm begins by performing a *K*-means clustering operation on the projection of the original gene expression matrix, **X**_*n*×*d*_, to the top *K*−1 principal directions. The number of initial clusters *K* is set to a sufficiently large value, say 30, to ensure most cell types will be captured. Once the initial clusters are determined, we take two subsets (*Y*_*i*_,*Y*_*j*_) that originate from a pair of clusters (*i*,*j*) respectively, and calculate the probability for those observations to be merged together, *p*({*Y*_*i*_,*Y*_*j*_}|*μ*_*ij*_,*Σ*_*ij*_). We assume that the probability density function is a multivariate Gaussian with mean and covariance matrix given by:

(1) $$ \begin{aligned} \mu_{ij} & = \frac{n_{i}}{n_{i}+n_{j}} \mu_{i} + \frac{n_{j}}{n_{i}+n_{j}} \mu_{j},\\ \Sigma_{ij} & = \frac{n_{i}}{n_{i}+n_{j}} \Sigma_{i} + \frac{n_{j}}{n_{i}+n_{j}} \Sigma_{j}, \end{aligned}  $$

where (*n*_*i*_,*n*_*j*_), (*μ*_*i*_,*μ*_*j*_) and (*Σ*_*i*_,*Σ*_*j*_) denote the sizes, centroids and covariances of the clusters *i* and *j* respectively. We repeat this for all possible pairs (*i*,*j*). We then choose to merge two clusters by either (i) picking the pair that has the highest probability or (ii) sampling a pair of clusters to merge in proportion to their (normalised) merged probabilities. The number of clusters will now decrease to *K*−1. We then project the data matrix on to the first *K*−2 principal directions, i.e. removing the (*K*−1)-st principal component that explains the lowest degree of variance in the data, removing this dimension from the existing cluster centroids and covariance matrices.

The above clustering operation is then repeated so that after every merge operation we remove a principal direction until only a single cluster remains. If sampling-based merge operations are used, the whole process can be repeated to obtain a number of alternative clusterings. This will be useful for assessing the stability of the clustering results. Algorithm 1 gives a pseudo-code description.



**Fig. 2:**
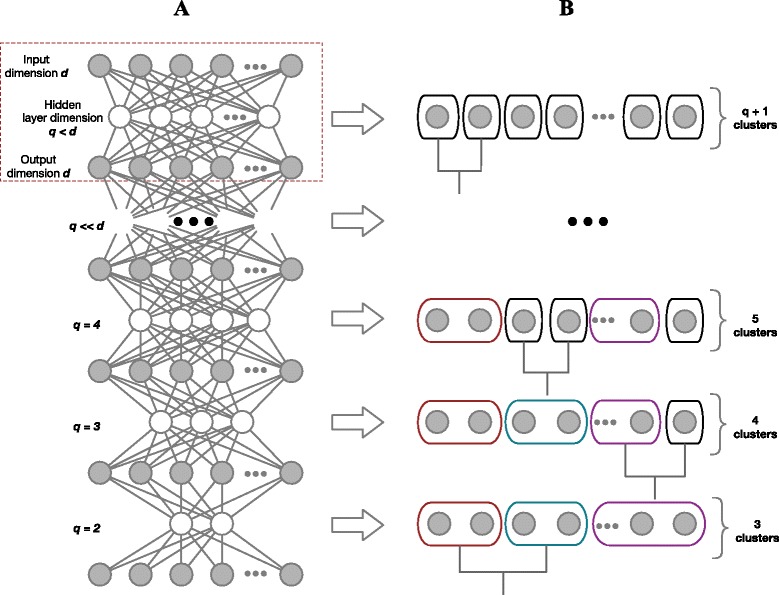
Method illustration using an autoencoder network. Clustering is applied to the data representation at each linear hidden layer. If there are *K*−1 linear hidden units, the data is projected into a subspace spanned by the top *K*−1 principal components. Consistency between the clusterings at each layer is maintained by enforcing a hierarchical constraint. **a** Graphical interpretation of an autoencoder network(s). **b** Corresponding hierarchical structure

Figure 2 gives an illustration of our method using an autoencoder representation. Autoencoders are feedforward neural networks that accept *d*-dimensional input data and report *d*-dimensional output data. The *d* nodes in the input and output network layers are connected via one or more *hidden* layers. Data transformations are applied between each layer of the network. If a hidden layer has fewer nodes than its predecessor then the information from the previous layer in the autoencoder network is forced into a lower dimensional form hence performing dimensionality reduction. Each hidden layer encodes a reduced dimensional representation of the input data. In an autoencoder, the parameters governing the data transformations between the layers are fitted to minimise the mean-squared error between the original input data and the output representation. It can be shown that, when using linear transformations, the optimal autoencoder is equivalent to doing principal components analysis [29]. Using this analogy it is now clear that *pcaReduce* can be seen as performing hierarchical clustering on the different hidden layers of a linear autoencoder network linking different clustering structures to different hidden layer representations of the input data. This analogy is useful to explain our algorithm and why we remove one principal component after each merge operation. We are in fact maintaining clustering consistency across the hidden layer representations of the input data in the autoencoder network.

## Results

To demonstrate the performance of *pcaReduce* method, we considered two single cell RNA-seq dataset examples. The first contains a collection of cells originating from diverse biological tissues [30]; and the second dataset the mouse sensory neuronal cells [27] discussed in the Introduction. These were selected as they contained pre-existing hierarchical cluster structures that can be used to assess unsupervised algorithmic performance. Here we show that *pcaReduce* can be applied to re-capture the known cellular hierarchies and we compare to other statistical techniques, which are commonly applied to address similar cell sub-typing problems. Below, all examples were implemented using the free statistical computing platform R (https://www.r-project.org).

### Cells from disparate tissues

**Fig. 3:**
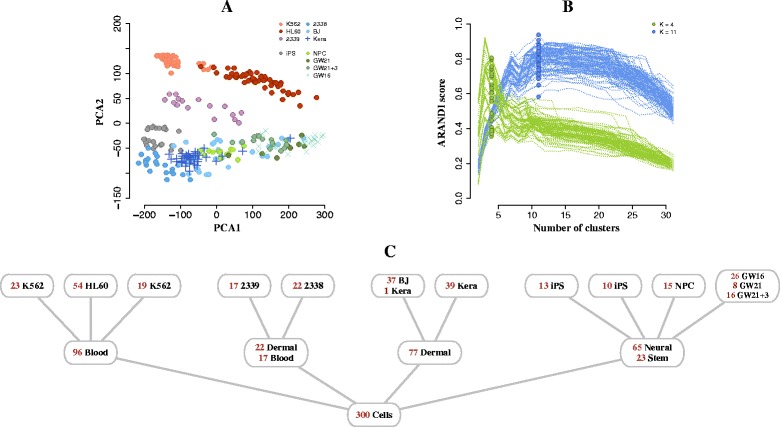
Application of *pcaReduce* to single cell RNA sequencing of 11 cell lines. **a** Projection of the data on to the first two principal components. **b** Performance of *pcaReduce*, the horizontal axis corresponds to a level in the hierarchical cluster structure reported by *pcaReduce*, the vertical axis show the Adjusted Rand Index (ARANDI) score between the tissue-level (*green*) and cell-line level labels (in *blue*) and the clustering reported by each level of the hierarchical clustering of *pcaReduce*. Each line correspond to a single run of *pcaReduce* using probabilistic sampling. **c** The most probable cellular hierarchy identified using *pcaReduce*

We obtained single cell RNA-seq dataset [30] for 300 cells whose transcriptional measurements were taken across 8,686 genes (see Additional file 1, Section A for further details on data preparation). The data were derived from 11 cell types: K562 – myeloid (chronic leukemia), HL60 – myeloid (acute leukemia), CRL-2339 – lymphoblastoid; iPS – pluripotent; CRL-2338 – epithelial, BJ – fibroblast (from human foreskin), Kera – foreskin keratinocyte; NPC – neural progenitor cells, GW(16, 21, 21+3) – gestational week (16,21, 21+3 weeks), fetal cortex (see Fig. 3a). In addition, as specified in the original study by [30], these cell types could also be grouped into four disparate tissue sources: blood, stem, skin and neural tissues. We refer to these as the *cell line-level* and *tissue-level* classifications respectively and use these as ground-truth classes in our performance assessment; i.e. we will focus on data partitions into *K*=11 and *K*=4 clusters.

We applied *pcaReduce* to this dataset to construct a hierarchical clustering of cells. First, we initially projected the data into the subspace spanned by the first 30 principal components following a PCA and performed an initial *K*-means clustering to get initial cluster assignments (using *K*=31 clusters) [31]. After this, we applied different merging strategies to construct the cellular hierarchies: first, when merging is performed based on the most probable cluster merge value (see (i) in Algorithm 1) and, secondly, when merge candidates are probabilistically sampled (see (ii) in Algorithmic overview section). The former gives a single hierarchical clustering whilst the latter can give a range of candidates hierarchies based on repeated sampling.

We compared the hierarchical clustering given by *pcaReduce* for levels *K*=4 and *K*=11 to the *true* cell line and tissue level classifications respectively using the Adjusted Rand Index [32]. Note that, in Fig. 3a, the projection of the eleven cell lines in two-dimensional principal component space cannot be separated into distinct groups. It is only possible to do this in higher dimensional representations. Figure 3b illustrates the performance of *pcaReduce* using the sampling-based merge operation where each line corresponds to a single run of the method. Although, *pcaReduce* has no knowledge of the true number of cell line or tissue labels, the correspondence between the hierarchical clustering output of *pcaReduce* and the true classification peaks at around levels 4 and 11 of the hierarchies respectively which it discovers without any prior knowledge.

In order to gain an understanding of the misclassifications, we looked specifically at the most probable hierarchical structure identified using *pcaReduce* (Fig. 3c). Compared to the known cell line and tissue labels (see Additional file 1: Figure S1), the 11-cluster structure given by *pcaReduce* did not fully differentiate the 11 cell types. This is not unsurprising since the 11 cell types included a set of three maturing neural cell types (GW16, GW21 and GW21+3) that are highly related. Interestingly, *pcaReduce* grouped these cell types together, which is not an entirely inappropriate operation since the expression variation between the maturing neural cells maybe relatively low compared to unrelated cell types. There was also some class splitting, for example, two sub-groups of K562 cells were identified. Figure 3a qualitatively indicates that this may make sense as some K562 cells were closer in overall expression to HL60 cells than other K562 cells.

At the 4-cluster level the assignments given by *pcaReduce* gave some interesting group structures. The ground truth tissue-level classification assumed the existence of blood, neural, dermal and stem cell types but *pcaReduce* identified that the CRL-2338 and CRL-2339 cell lines should form a group. This is interesting as CRL-2338 is a cell line derived from a primary stage IIA, grade 3 invasive ductal carcinoma and CRL-2339 is a B lymphoblastoid cell line initiated from peripheral blood lymphocytes from the same patient. Pluripotent stem cells (iPS) were also grouped by *pcaReduce* with neural progenitor cells (NPC) which is also reasonable if we consider this a stem cell-like group. Overall, whilst *pcaReduce* did not give a 4-cluster classification that was identical to the original tissue classifications [30], the output produced are not nonsensical. In comparison, the output of standard hierarchical clustering failed to separate the cells both at the cell line and tissue level into any obvious structure (see Additional file 1: Figure S1).

**Fig. 4:**
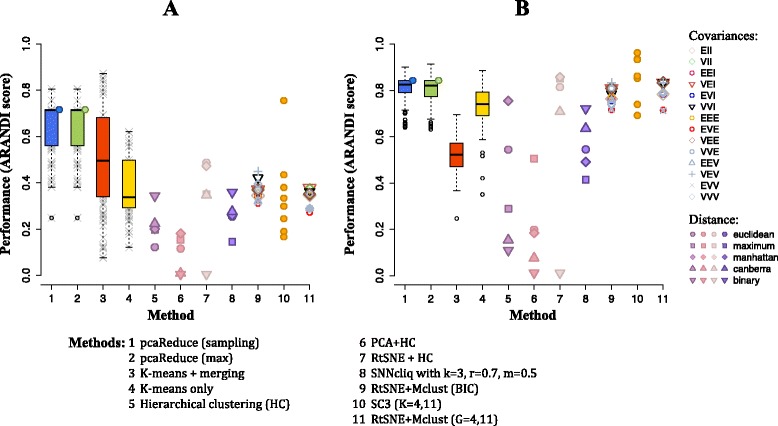
Performance comparison on cell line data. Classification performance against known **a** tissue-level and **b** cell-line level labels. All points and boxplots illustrate performance relative to the benchmark (Method 11) measured as ARANDI score. Numbers 1−11 correspond to clustering methods in table below. Blue and green circles for Methods 1-2 illustrate consensus clustering of 100 runs of *pcaReduce* algorithm with sampling and max merging settings respectively. Each point for Method 10 (SC3) corresponds to a different range of the parameter *d*. Further details can be found in Additional file 1: Figure S3

In order to fully assess the performance of *pcaReduce*, we compared it to a set of alternative approaches (see Fig. 4). This includes popular methods such as: *K*-means, hierarchical clustering (HC), Mclust – mixture modelling for model-based clustering [33] combined with tSNE – a nonlinear dimensionality reduction/visualisation technique [22]; and recent single cell methodology – SNN-Cliq, which determines similarities between cells based on a shared nearest neighbours algorithm and performs single cell clustering using a graph-theoretical approach [25], and SC3 which uses spectral transformations of a cell-to-cell distance matrix followed by *k*-means and consensus-based clustering [26]. Details regarding all parameters and running specifications for each clustering approach are summarised in Additional file 1.

Figure 4 shows the relative performance of the different approaches. The score of t-SNE/Mclust (Method 11) was used as a benchmark and the true number of cell lines was given as a known parameter. The t-SNE algorithm is a frequently used non-linear dimensionality reduction technique for single cell expression analysis and Mclust is a well known and popular clustering algorithm (based on Gaussian mixture models). The combination provides considerable considering flexibility. When we compared the classifications at *K*=11 of the various hierarchical clustering methods, including our own *pcaReduce*, we found that the standard hierarchical clustering approaches did less well than *pcaReduce*. For example, Method 3 uses the same initial *K*-means cluster initialisation as *pcaReduce* and cluster merging criterion but does not use different data representations by removing principal components after each merge. This subtle alteration in methodology appears to make a fundamental difference in performance. Method 6 uses hierarchical clustering applied to the same initial principal component projection of the data as *pcaReduce* but performance is again low and highly dependent on distance measure used for the clustering (see Additional file 1: Figure S4).

Whilst some methods had comparable performance to *pcaReduce* in terms of capturing the cell line level classifications, their performance diminished for the tissue-level ones. Here, the benchmark used was Method 11 when the number of clusters (4) was given as an input parameter. Despite this advantage it failed to identify any clustering structure even closely resembling the ground truth we are using. Similarly, Method 7 uses hierarchical clustering applied to a t-SNE projection of the data, this had a reasonable ARANDI score for the cell line level classification but when the clusters were merged into 4 groups these had no correspondence to the ground truth. SC3 was able to achieve comparable performance to *pcaReduce* for specific range of values for a parameter *d* – the number of eigenvectors retained following the spectral transformation of the cell-to-cell distance matrix. However, the range of *d* that gave greatest concordance was *not* the default setting (0.04*N*<*d*<0.07*N*), see Additional file 1: Figure S2. Overall, *pcaReduce* gave consistently provide clustering results that were closest to the ground truth (see ARANDI score in Fig. 4) for both the cell line and tissue classifications. Its performance suggests that the gradual use of successively reduced dimensional representations of the data helps to merge clusters together in a sensible way.

Note, there is stochasticity in the clustering structures produced by *pcaReduce* due to the random initialisation provided by the *K*-means algorithm and probabilistic merge steps. Interestingly, motivated by the use of consensus clustering in SC3, we applied an ensemble clustering method across the *pcaReduce* clustering structures (see Additional file 1 for details of the methods used), the consensus clustering structure achieves a high level of concordance with the cell line and tissue level classification. Finally further details regarding sensitivity in initial selection of *q* – the initial number of clusters used – is shown in Additional file 1: Figure S3).

### Mouse neuronal cells

**Fig. 5:**
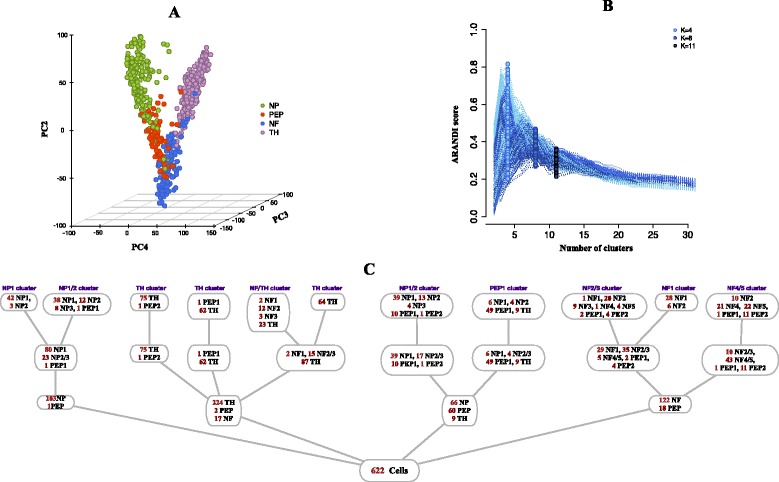
Application to single cell mouse neuronal data. **a** Data projected on to PC2-4 for visualisation and coloured by the four major neuronal cell types. **b** Clustering performance of *pcaReduce*. **c** Cellular hierarchy identified using *pcaReduce*, further details are given in Additional file 1: Figure S4

We next returned to the mouse neuronal cell dataset discussed in the Introduction that contains measurements across 25,334 genes [27]. The study classified cells according to four principle neuronal groups: non-peptidergic nociceptor cells (NP), peptidergic nociceptor cells (PEP), neurofilament containing cells (NF), and tyrosine hydroxylase containing cells (TH) (Fig. 5a). In addition to this, it was suggested that the NP, PEP and NF cells possessed further subtypes (Fig. 1b, c). We now examined whether *pcaReduce* could recover these three layers of clustering structures within its hierarchical output without the use of marker genes.

We applied *pcaReduce* and computed the correspondence between its 4-, 8- and 11-cluster structures and those in the original study. Figure 5b shows that the absolute classification accuracy was relatively low compared to the previous cell line experiment. This is unsurprising as the four pre-dominant neural cell groups form a complex cluster pattern in the subspace spanned by PC2-4 (see Fig. 5a) and would be hard to segregate in an entirely unsupervised way as we propose. This is especially evident from PCA plots summarised in Additional file 1: Figure S5, where we plot pairwise combinations of various principle components and highlight cells that should correspond to neuronal subtypes: NF3 and NF4 (lower-left) and NP2 and NP3 (upper-right).

The performance of *pcaReduce* generally outperformed the other approaches we tried across the three clustering structures (see Additional file 1: Figure S6) except for Method 11 (t-SNE + Mclust), which used the true number of clusters by default and acts as an artificial benchmark, and SC3 (Method 12). Interestingly, classification performance was again increased by applying consensus clustering across the *pcaReduce* sampled clustering structures. Although this had greater effect for the 4-cluster structures than the 8- and 11-clusters. In the case of SC3, the classification performance was sensitive to the choice of the number of eigenvectors (*d*) used in the spectral transformation step (see Additional file 1: Figure S7). Strong classification performance was obtained using the default range of *d* but we note that this range was chosen based on optimizing against a number of single cell datasets and their ground truth classifications including this mouse neuronal dataset [26]. Outside of this default range, performance varied and could be similar to the levels obtained by other methods (Additional file 1: Figure S7).

**Fig. 6:**
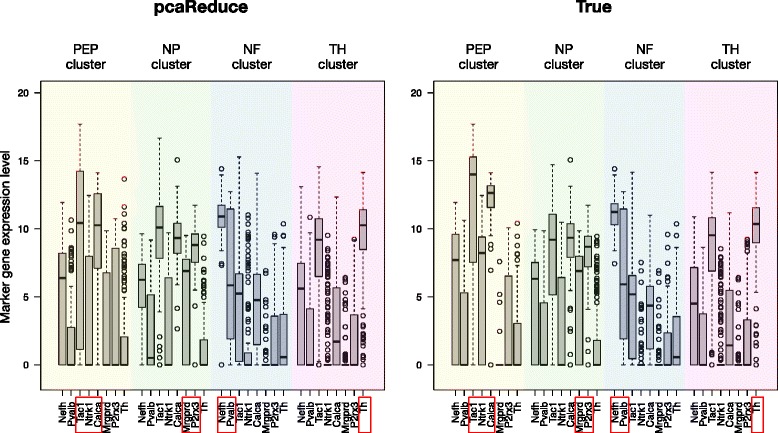
Performance comparison on mouse neuronal data. Boxplots illustrate the expression levels of marker genes that define four major neuronal classes. **a** Illustrates results obtained using *pcaReduce* algorithm, whereas (**b**) illustrate the ground truth. The information about marker genes was obtained from [27]

Using the most probable structure given by *pcaReduce*, we noted that the three groups of the four top level groups are predominantly dominated by NP, TH and NF cells respectively (Fig. 5c) matching groups in the classification by [27]. Marker gene expression patterns (obtained from [27]) for these three groups also corresponded to those found in the original study confirming their identities (Fig. 6). The main source of discordance comes from 66 NP cells being assigned to the same group as 60 PEP cells giving a combined group of these cells (NP/PEP) not present in the original classification. When we examined the expression of the marker genes, we discovered that the expression of these genes was strikingly similar between the NP and PEP groups found by [27] with the only major difference being complete zero expression of *Mrgprd* in the PEP group whilst only some NP cells show zero expression for this gene. Therefore it is perhaps unsurprising that cells from these two groups were merged by *pcaReduce*. Interestingly, the PEP and NP cells correspond to sub-classes of nociceptors (peptidergic and nonpeptidergic respectively). This combined NP/PEP groups does subsequently become partitioned as the number of clusters was allowed to increase into subgroups dominated by NP and PEP cells respectively. Note that the use of t-SNE – a non-linear dimensionality reduction technique – did not well-separate the four groups either (Additional file 1: Figure S8), and it would not be obvious, without known markers, how to delineate each group.

At the 11-cluster level *pcaReduce* identified multiple subgroups of TH cells that had high *Th* gene expression but possessed different patterns of expression in other marker genes (Additional file 1: Figure S9). In contrast, the original study [27] only possessed one Th group. This difference alone drives much of the classification discordance between *pcaReduce* and the original classes but this discordance may not be an “error” but simply a *different* choice of clustering compatible with the same data. Further differences are driven by the combined NP-PEP cluster generated by *pcaReduce* that is then propagated down the hierarchical structure. Finally, the decomposition of the NF cluster by *pcaReduce*, splits the NF group into three subgroups with striking similarity to the NF1, NF2/3 and NF4/5 groups in [27].

## Discussion

In this paper we have presented an unsupervised hierarchical clustering approach for the identification of putative cell sub-populations from single-cell transcriptomics profiles. Clustering occurs in a linearly transformed subspace obtained from principal component directions and, at each level of our hierarchical clustering structure, the similarity between clusters is measured in subspaces of *decreasing* dimensionality by discarding principal directions as the number of clusters decreases. In doing so, we presume that the variation contained in the first principal components corresponds to the features of broad cellular classes, whilst fine-scale variation in lower principal directions correspond to the features of detailed cellular sub-structure. We also implicitly assume that the clusters are separable in the principal component subspaces. For data sets where this does not apply, it maybe possible to perform an non-linear transformation of the data first, before applying *pcaReduce* although this would lead to the loss of simple interpretation for the principal components attached to each merge in the hierarchical clustering.

We applied this technique to two illustrative single cell datasets from the recent literature and showed that, compared to a variety of existing clustering tools, our approach was able to better recapitulate pre-existing cluster structures across both – broad *and* detailed cellular states; further, this was achieved *simultaneously* in a hierarchical fashion. Interestingly when we specifically compared *pcaReduce* to related variants of the method, which did not use successively reduced dimensional representations, we showed that clustering performance was worse for these alternate approaches. Intuitively, this might be expected since the “distances” between cells in high-dimensional spaces can be unstable depending on the measure used. By using low-dimensional data representations to describe low-complexity cluster structures we reduce the possibility that variability in higher dimensions impacts on clustering performance. In summary, the key advance of our method is that we provide consistent clustering across different reduced dimension representations of the data. This is important because the choice of reduced dimension representation will allow a different number of clusters or cell types to be resolved. Although we cannot define how many *real* cell types exists nor which representation are optimal for finding them, *pcaReduce* will return consistent clustering across the different representations such that, for example, a 4-cluster structure will always be related to a 5-cluster representation and that the extra cluster is related to the extra dimension of information introduced.

Benchmarking our algorithm and related methods is extraordinarily difficult since there is a lack realistic, gold standard data sets with known cell types. The disparate tissue data used in our first application has known cell types but could be considered artificial in that the constituent cell populations are derived from completely independent cell lines and have a different genetic background. Furthermore, the tissue-level categorisation we used as our ground-truth is potentially arbitrary. Alternative groupings of the cell lines could be made that are qualitatively sensible. The mouse neuronal data is more representative of the type of data that our method targets where the main is to explore expression heterogeneity against a fixed genetic background but in such situations the *true* cell types are unknown and, as our analysis has shown, there is potential subjectivity in how we define novel cell types. Other data sets, such as the five primary glioblastomas in [12], could be used by defining a cell type as the glioblastoma from which the cells are derived but again this would be examining expression variability against a variable genetic background. Unfortunately, as yet, there are no realistic simulation algorithms that can generate suitable high-dimensional single cell gene expression data.

## Conclusions

We conclude by remarking that the absolute performance of ours and other techniques can be rather limited in an unsupervised setting, and further research is required to combine local and global feature selection alongside clustering/classification techniques is necessary in order to better identify real cell types and states. Cluster validation with single cell data is also challenging since there are often no independent means of establishing the validity of computationally derived cell types. We therefore emphasise that such tools are primarily exploratory devices and more extensive functional validation is required.

Finally, we previously described our hierarchical clustering approach within an autoencoder network framework. This analogy offers the possibility for further methodological extensions. Our implementation uses principal components analysis which in an equivalent autoencoder representations corresponds to using linear transformation between hidden layers. However, non-linear transformations can also be applied leading to give greater flexibility and improved dimensionality reduction properties. We are currently exploring this feature and aim to implement it in the next version of *pcaReduce*.

## Availability and supporting data

The details of the data sets supporting the results of this article are detailed in Additional file 1. The R source code for *pcaReduce* is freely available from our Github repository: https://github.com/JustinaZ/pcaReduce.

## Additional file

Additional file 1 Supplementary Information. A pdf file that contains additional information and results omitted from the main paper. (PDF 2570 kb)
