# Supplementary material for: pcaReduce: hierarchical clustering of single cell transcriptional profiles
Source: BMC Bioinformatics. 2016 Mar 22;17:140. doi: 10.1186/s12859-016-0984-y (PMC4802652; doi:10.1186/s12859-016-0984-y)
Supplement: Additional file 1 — Supplementary Information. A pdf file that contains additional information and results omitted from the main paper. (PDF 2570 kb) [file 12859_2016_984_MOESM1_ESM.pdf]

List of Figures

S1 Comparison of *pcaReduce* and standard hierarchical clustering on the disparate tissues data. 5
S2 Variability of sc3 clustering structures on Pollen data. 6
S3 Sensitivity of *pcaReduce* output to initial q-values. 7
S4 Hierarchical classification of mouse neuronal cells. 8
S5 Separability of mouse neuronal cell types in principal component space. 9
S6 Comparison of classification performance on mouse neuronal cells 10
S7 Variability of SC3 clustering structures on mouse neuronal data. 11
S8 t-SNE visualisation of mouse neuronal data. 11
S9 Marker gene expression levels across 11 neuronal clusters identified using the *pcaReduce* algorithm. 12

Additional File

Note on low level single cell data processing and gene expression matrix preparation

Cells from disparate tissues.

We have downloaded RNA-seq dataset from NCBI repository (`http://www.ncbi.nlm.nih.gov`) under accession number SRP041736, which contains transcriptional profiles of 347 singles cells. Next, we have converted the Sequence Read Archive (SRA) files into *fastq* files using SRA Toolkit (`http://www.ncbi.nlm.nih.gov/sra/`), and used TopHat-2.0.12 [7] to perform genomic mapping of pair-end reads to the latest human reference genome GRCH38 (`http://www.ensembl.org/info/data/ftp/index.html`). We have used R package called *Rsubread* [4] to assign mapped sequencing reads to genomic features, i.e. to perform transcript counting, which was achieved using function *featureCounts*.

In order to construct a gene expression matrix for higher level analysis, we have performed basic cell and gene filtering: (a) From 347 samples we have focused on a subset of 301 cells (a subset without ERCC validation cells and bulk sample), which were used in the main study by Pollen et al. [5]. In addition, we have removed one cell that had 0 expression levels across all genes, this left us with a 300 cells in total. (b) For gene filtering we have used R package called *edgeR* [6], we kept those genes that fulfilled at least 100 counts per million (cpm) in at least 10 samples; this left us with 8686 genes in total. Lastly, we have transformed gene expression counts to a logarithmic values; more precisely, values x_{ij} in matrix X were obtained by $\log_2(x^0_{ij} + 1)$, where x^0_{ij} are read counts of a gene.

Mouse neuronal cells. We have downloaded readily pre-processed dataset from the website `http://linnarssonlab.org/drg/`. For the main study, we focused on 622 cells that were classified as neurones

[8].

Note on K-means algorithm

Within *pcaReduce* package K-means method operates using fixed settings – the default algorithm is set to be Hartigan-Wong algorithm [2], with a number of several random starts (we fix it to be $nstart = 20$); the latter option attempts to address the sensitivity in selecting initial centroids.

Note on consensus *pcaReduce*

As the output of *pcaReduce* is stochastic and dependent on the initial k-means initialisation and probabilistic merge steps, each run of the algorithm may produce varying results. In order to obtain a consensus, we ran *pcaReduce* algorithm 100 times with sampling as a merging criterion and then used the ensemble clustering methods implemented in the R package *"clue"*. We used the built-in method "SE", based on least squares Euclidean consensus partitions. We used the following control parameters: K – the number of classes – to be the true number of clusters $(4, 8, 11$ – depending on example used); and $nruns$ – the number of runs to be performed – to be 50. Similar consensus approch was taken for *pcaReduce* algorithm with max merging criterion.

Note on clustering tool comparison

- **K-means and merging**. Using PCA we project initial dataset to $q = 30$ and partition it into $K = 31$ clusters using K-means. Next, we merge two clusters together using sampling as a merging criterion. However, in the next step instead of dropping off the last dimension (i.e. instead of $q \longleftarrow q - 1$ in Algorithm 1 in main paper), we keep $q = 30$ fixed, and continue merging clusters as described before. We repeat these steps 100 times. This small method alteration illustrates the importance of gradual dimensionality reduction, as it affects the *pcaReduce* performance quality on both datasets (see Method 3, averaged ARANDI scores in Figures 4 and 6 in main paper).

- **K-means only**. We ran the K-means clustering algorithm for a full dataset 100 times without prior dimensionality reduction step; using either the true number of clusters, e.g. for the Pollen data set $K = 4$ and $K = 11$, and computed the ARANDI scores between these clusterings and known cellular labels (e.g. see Method 4 in Figure 4 A and B respectively).

- **Hierarchical clustering**. We ran the hierarchical clustering algorithm with all possible distance measures: Euclidean, maximum, Manhattan, Canberra, and binary. Each time we cut hierarchical tree at e.g. $K = 4$ and $K = 11$ and compute ARANDI scores between these clusterings and known cellular labels (e.g. see Method 5 in Figure 4 A and B respectively).

- **PCA followed by Hierarchical clustering**. Using PCA we project initial dataset to $q = 30$ and run hierarchical clustering algorithm with all possible distance measures. Each time we cut hierarchical tree at e.g. $K = 4$ and $K = 11$ and compute ARANDI scores between these clusterings and known cellular labels (e.g. see Method 6 in Figure 4 A and B respectively).

- **RtSNE followed by Hierarchical clustering**. Using RtSNE we project data on to two-dimensional space, we select the number of dimensions that should be retained in the initial PCA step to be 30 (the same as $q = 30$), and set perplexity parameter to be 30, further we use accuracy parameter to be default, $\theta = 0.5$. We ran the hierarchical clustering algorithm with a range of possible distance measures: Euclidean, maximum, Manhattan, Canberra, and binary. Each time we cut hierarchical tree at e.g. $K = 4$ and $K = 11$ and compute ARANDI scores between these clusterings and known cellular labels (e.g. see Method 7 in Figure 4 A and B respectively).

- **SNN-Cliq**. We ran the SNN-Cliq [11] tool on a full dataset using a range of possible distance measures: Euclidean, maximum, Manhattan, Canberra, and binary. We keep default settings, i.e. $k = 3$, which is the size of the nearest neighbours, $r = 0.7$, which is a parameter for finding quasi-cliques, and $m = 0.5$ – a merging parameter; the later two parameters affects cluster compositions. Next, we compute ARANDI score between true clusterings, $K = 4$ and $K = 11$, and clusterings determined by SNN-Cliq. (e.g. see Method 8 in Figure Figure 4 A and B respectively). It is worth noting that by examining various parameter k, r, m combinations, one potentially could achieve a greater agreement between estimated and known clusterings. However, this could also pose some difficulties in situations where cluster labels are unknown.

- **RtSNE in conjunction with Mclust**. We use t-SNE [10] (R package *Rtsne* [9]) to project dataset on to two-dimensional space, we select the number of dimensions that should be retained in the initial PCA step to be 30 (the same as $q = 30$), and set perplexity parameter to be 30, further we use accuracy parameter to be default, $\theta = 0.5$. Next we use model based clustering, Mclust [1], with all possible covariance models to cluster projected dataset. We use the following strategies:

  1. To determine the number clusters that best describes provided dataset, we use Bayesian information criterion (BIC). We test the number of mixture components in the range of $G = 1 : 31$. Next we compute ARANDI scores between the true cluster labels, $K = 4$ and $K = 11$, and labels identified with BIC (e.g. see Method 9 in Figure 4 A and B respectively).

  2. Alternatively, using Mclust we fit two finite mixture models with fixed number of mixture components, $G = 4$ and $G = 11$. Again we compute ARANDI scores between true clusterings and clusterings identified by Mclust (e.g. see method 10 in Figure 4 A and B respectively).

- **SC3**. R packages was obtained on 29/01/2016 from Bioconductor. We used function sc3 with

default parameters except for ks, which was set to $ks = 4, 11$ for the cell line data, and $ks = 4, 8, 11$ for mouse neuronal cells. We ran the sc3 algorithm with both default and custom values for the parameter d – the number of eigenvectors of the transformed distance matrix.

Note on starting q value

Here we explore the effects on selecting various starting values q. We select five different starting strategies, $q = 15, 20, 30, 50, 100$, and each time we run *pcaReduce* algorithm for 100 times. Figures S3 illustrates how clustering results depend on the initial choice of q value (otherwise largest K). We found that for this example $q = 30$ was a good choice and delivered data partition the most similar to the ground truth.

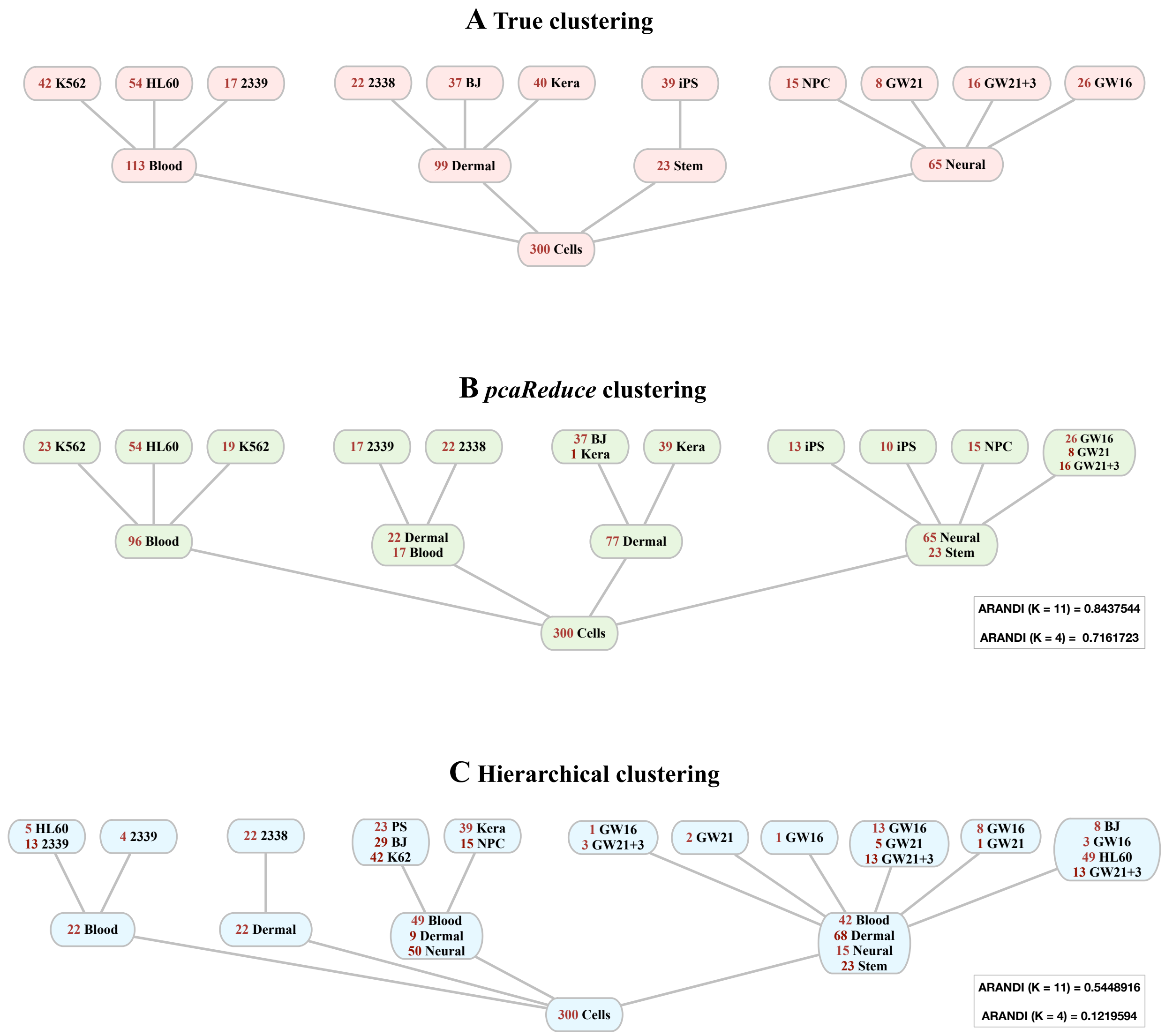

Figure S1: **Comparison of *pcaReduce* and hierarchical clustering results on the disparate tissues data.** (A) Hierarchical relationships between cells based on known cellular labels. (B) Hierarchical relationships between cells based on cellular labels identified by single run of *pcaReduce* under the most probable merging criterion. Also ARANDI scores between true clusterings, $K = 4$ and $K = 11$, and identified clusterings by *pcaReduce* are summarised in grey rectangles. (C) Hierarchical relationships between cells based on cellular labels identified by a standard hierarchical clustering (HC) algorithm; ARANDI scores between true clusterings, $K = 4$ and $K = 11$, and identified clusterings by HC are summarised in grey rectangles.

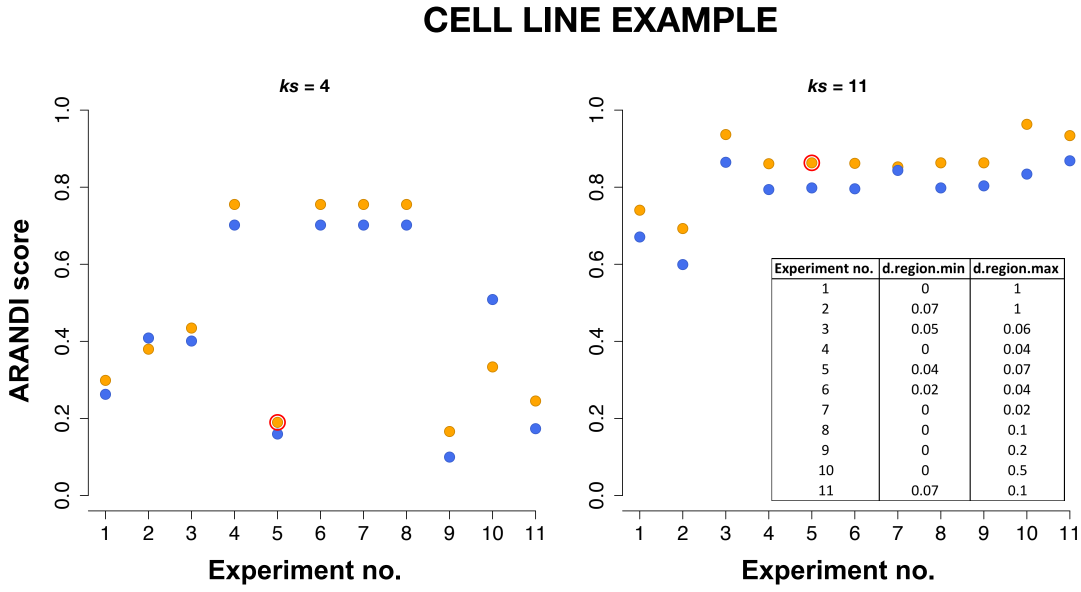

Experiment no.	d.region.min	d.region.max
1	0	1
2	0.07	1
3	0.05	0.06
4	0	0.04
5	0.04	0.07
6	0.02	0.04
7	0	0.02
8	0	0.1
9	0	0.2
10	0	0.5
11	0.07	0.1

Figure S2: **Variability of sc3 clustering structures** Plots illustrate SC3 [3] performance for different parameter ranges d given in the table (inset) for the tissue ($ks = 4$) and cell line ($ks = 11$) level classifications. Orange points show the ARANDI scores between the true clustering structure and those identified by sc3. Blue points show the ARANDI scores computed between the *pcaReduce* ensemble clusters and those of SC3. In red we highlight the scores obtained using the default d range used in SC3.

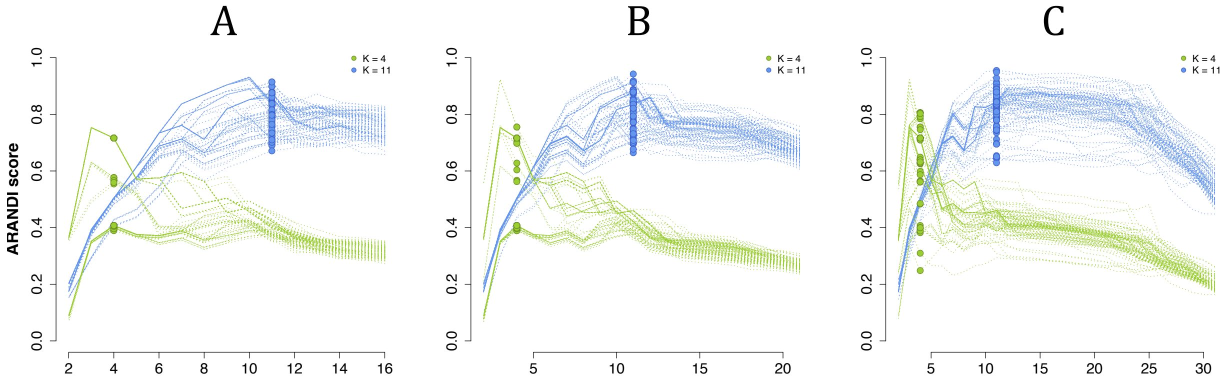

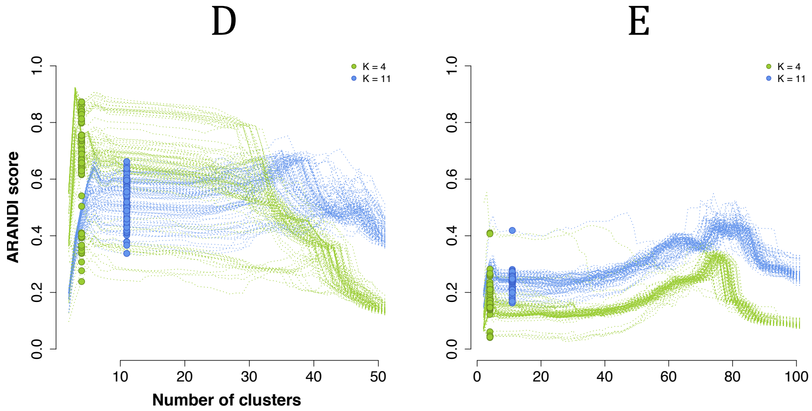

F

	K = 11	K = 4
q = 15	0.8044904	0.5781924
q = 20	0.8119908	0.6075658
q = 30	0.8209323	0.6649249
q = 50	0.5345074	0.6689931
q = 100	0.2379905	0.1751235

Figure S3: **Sensitivity of *pcaReduce* output to initial q-values.** (A) – (E) shows ARANDI scores for different parameter settings $q = 15, 20, 30, 50, 100$ respectively between the true clusterings, $K = 4, 11$ (green, blue), and 100 alternative clusterings identified by *pcaReduce* algorithm. Table (F) corresponds to the averaged ARANDI scores.

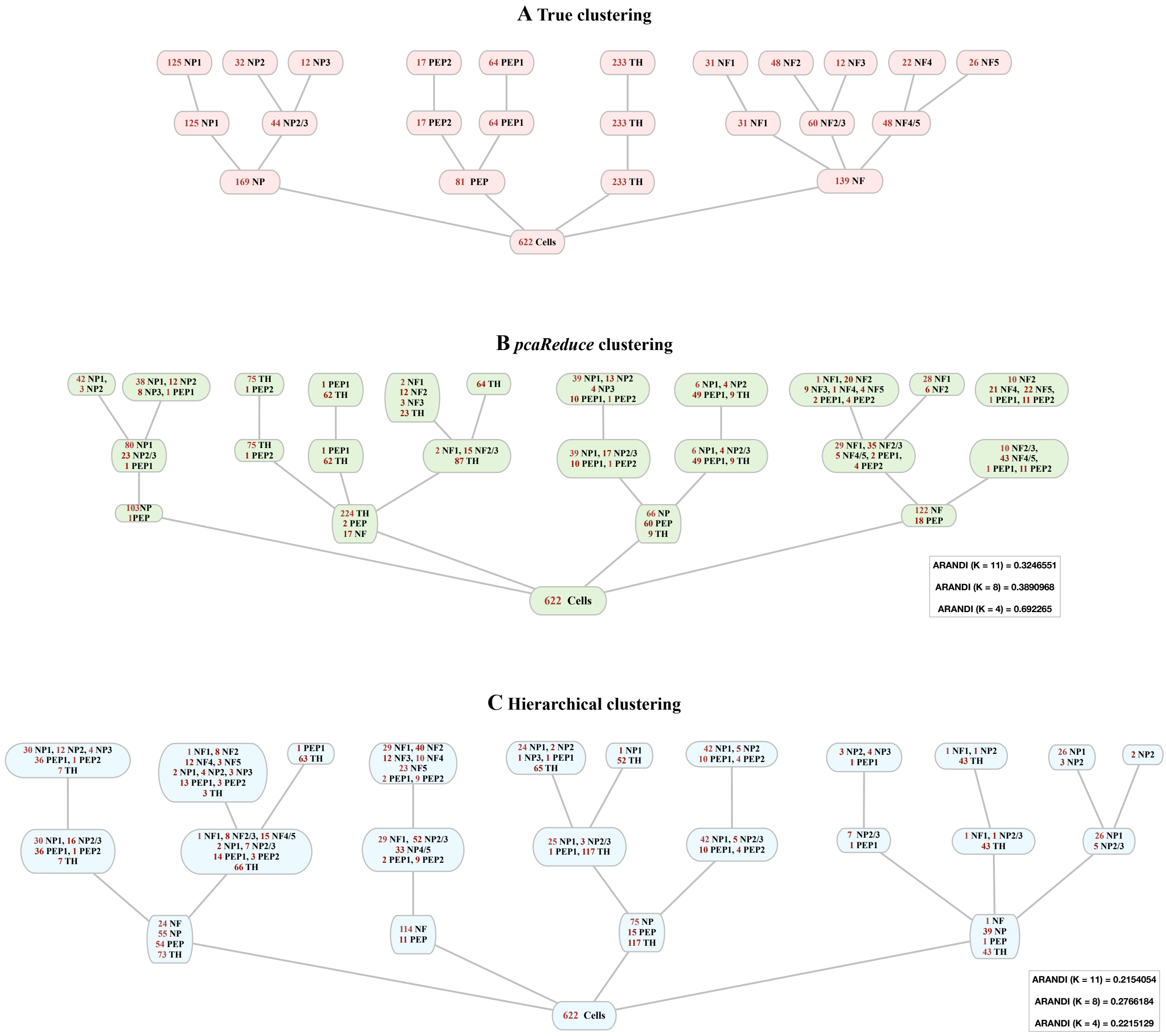

Figure S4: **Hierarchical classification of mouse neuronal cells.** (A) Hierarchical relationships between cells based on known cellular labels. (B) Hierarchical relationships between cells based on cellular labels identified by single run of *pcaReduce* under the most probable merging criterion. Also ARANDI scores between the true clusterings, $K = 4, 8, 11$, and identified clusterings by *pcaReduce* are summarised in grey rectangles. (C) Hierarchical relationships between cells based on cellular labels identified by a standard hierarchical clustering (HC) algorithm; ARANDI scores between true clusterings, $K = 4, 8, 11$, and identified clusterings by HC are summarised in grey rectangles.

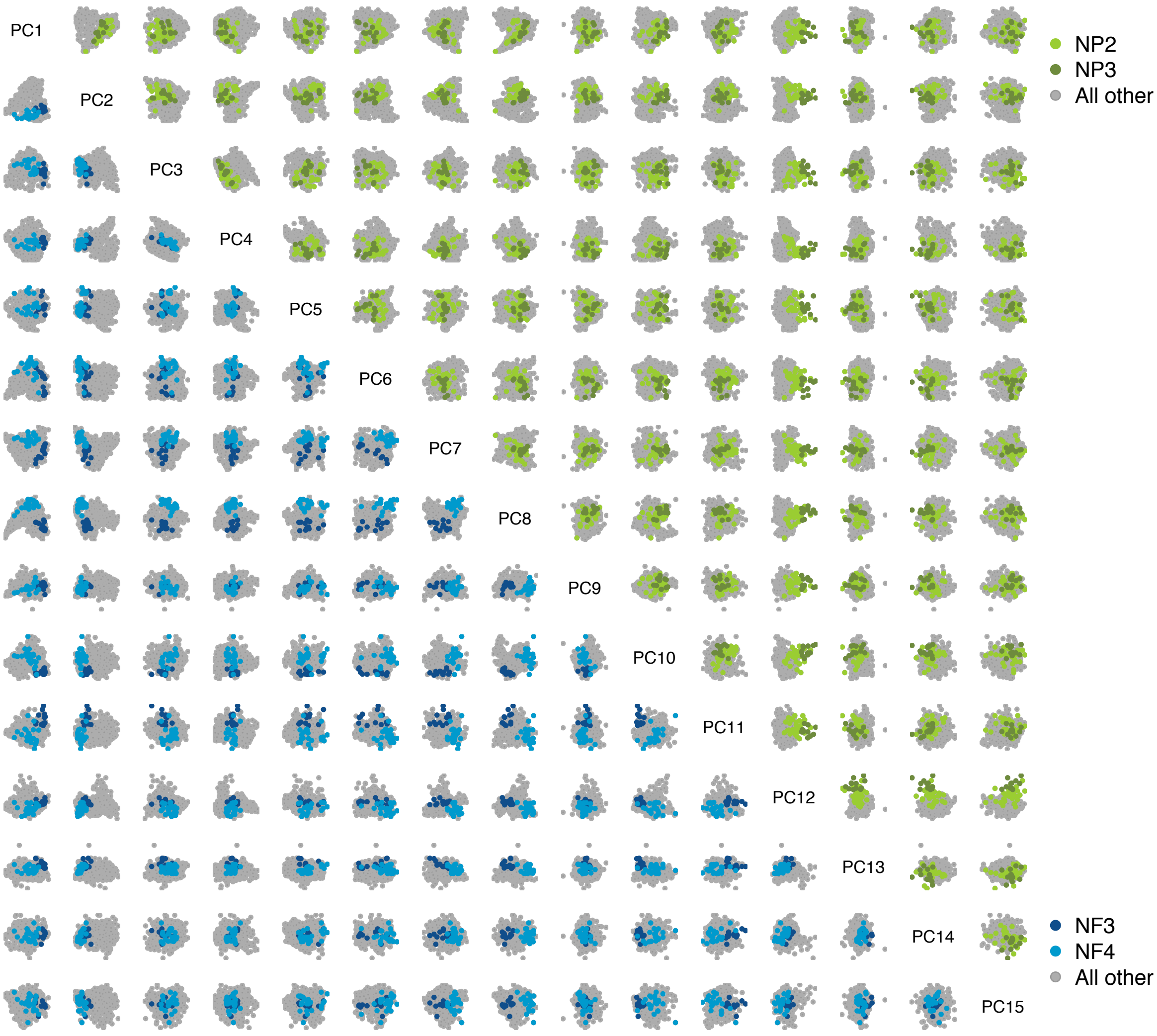

Figure S5: **Separability of mouse neuronal cell types in principal component space.** Scatterplots illustrate the first 15 principle directions. In the left triangle NF3 and NF4 cells are highlighted in blue and dark blue points. In the right triangle NP2 and NP3 cells are highlighted in green and dark green points.

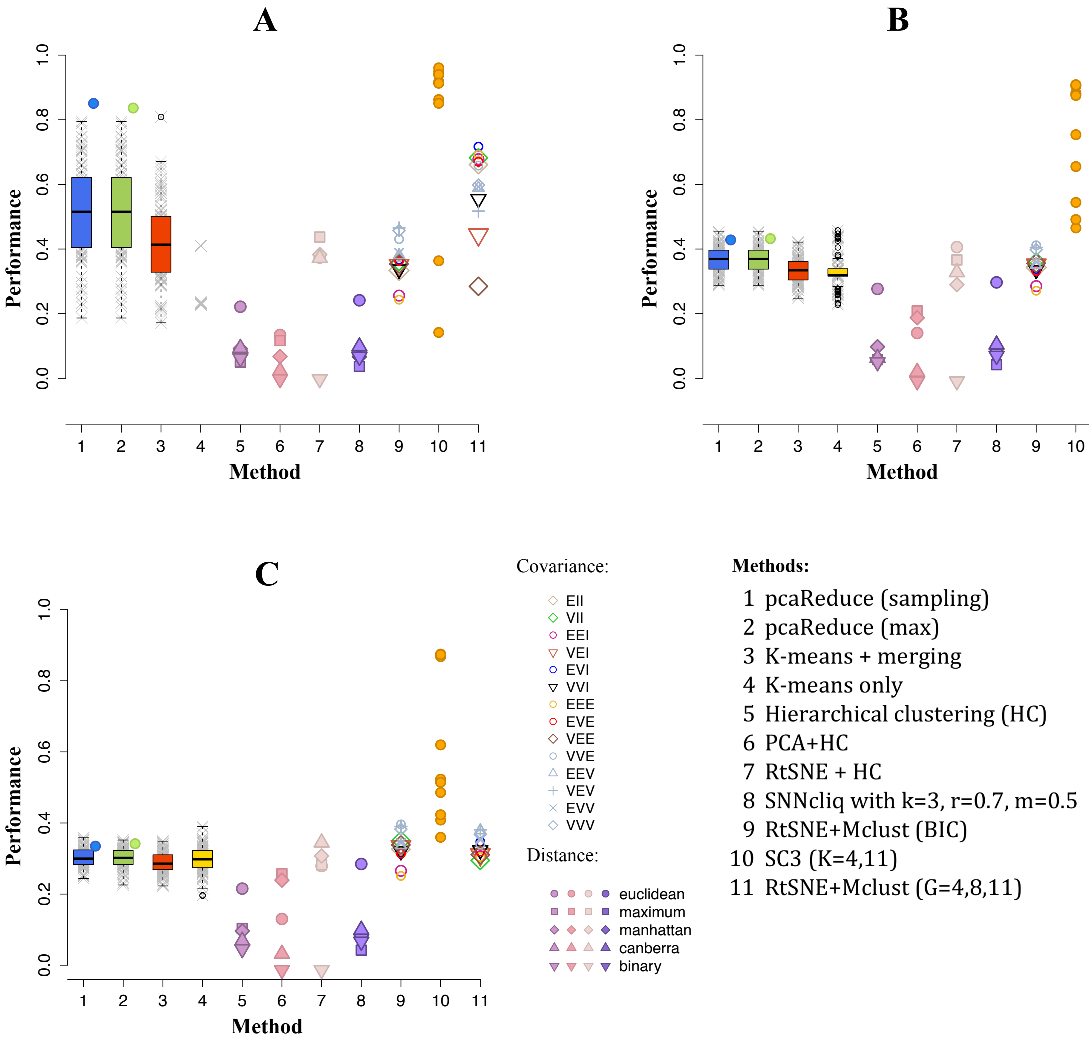

Figure S6: **Comparison of classification performance on mouse neuronal cells**. Boxplots 1–4 are based on 100 runs of each method, blue and green circles correspond to the outcome of consensus clustering of *pcaReduce* output across 100 runs with sampling and max merging settings respectively; methods 5–8 are evaluated based on all possible distance metrics; 9,11 based on all possible covariance structures, method 11 based on various parameter d ranges. (A) ARANDI score between true clustering, $K = 4$, and clusterings identified by Methods 1–11. (B) ARANDI score between the true clustering, $K = 8$, and clusterings identified by Methods 1–11. (C) ARANDI score between true clustering, $K = 11$, and clusterings identified by Methods 1–11.

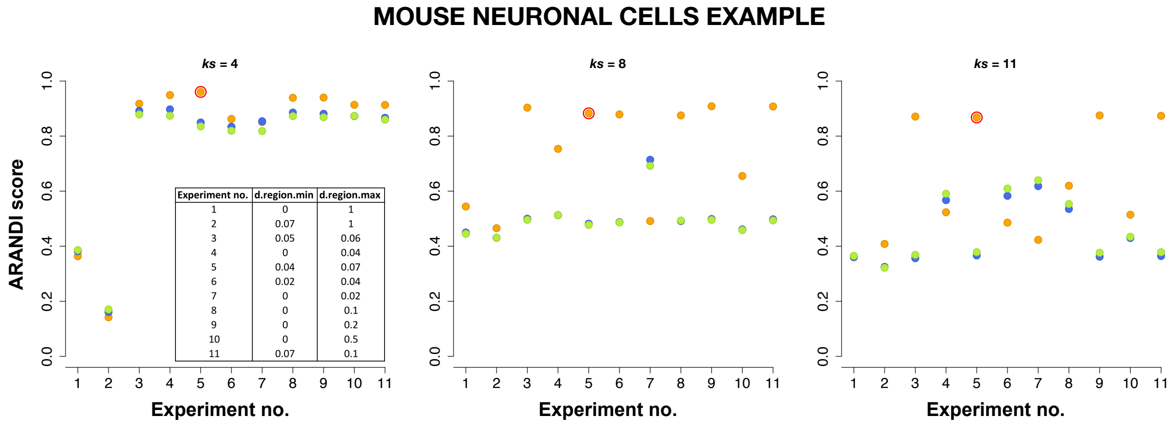

Figure S7: **Variability of SC3 clustering structures on mouse neuronal data.** Plots illustrate SC3 performance, where parameter ranges for d shown in the table (inset). Orange points show the ARANDI scores between the true clustering structure and those identified by sc3. Blue points show the ARANDI scores computed between the *pcaReduce* ensemble clusters and those of SC3. In red we highlight the scores obtained using the default d range used in SC3.

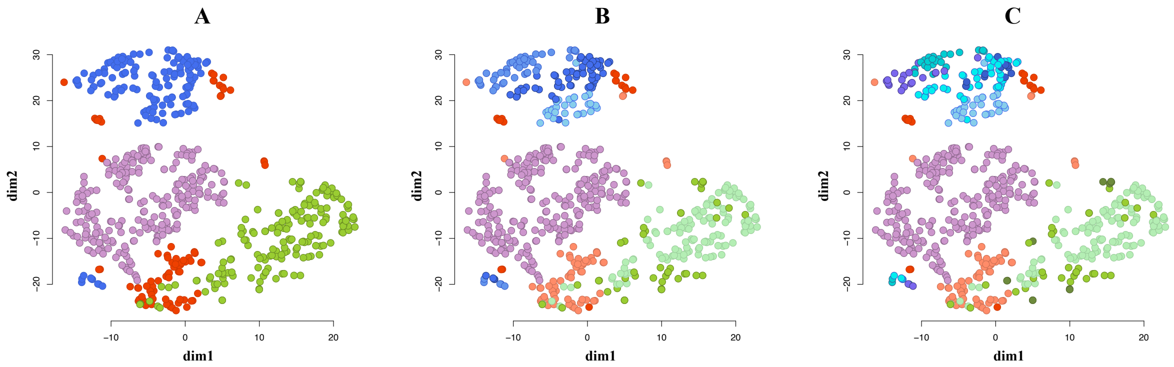

Figure S8: **RtSNE visualisation of the mouse neuronal data**. The data was projected on to a two-dimensional space, here cells are coloured based on the known cell type labels for: (A) 4, (B) 8, and (C) 11 clusters.

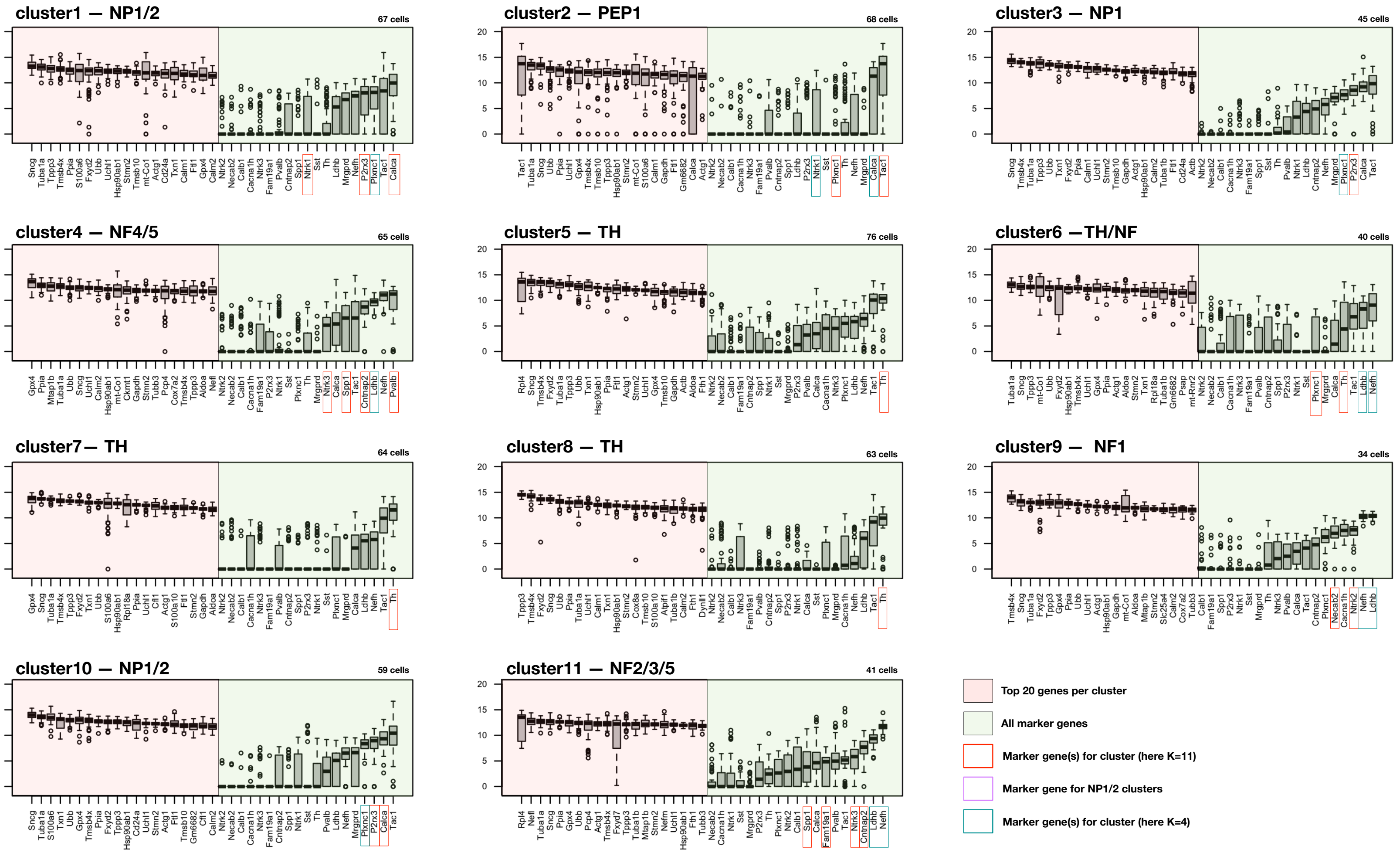

Information about marker genes is taken from [7], Figure 3

NF1	NF2	NF3	NF4	NF5	NP1	NP2	NP3	PEP1	PEP2	TH
Ntrk2(low)	Ntrk2(high)	Ntrk3	Ntrk3	Cntnap2	P2rx3	Ntrk1	Sst	Tac1	Fam19a1	Th
Necab2	Calb1	Fam19a1	Pvalb	Spp1		Calca	P2rx3	Ntrk1		
Cacna1h					Plxnc1			Calca		

Marker genes for major neuronal classes [7]

NF	PEP	NP	TH
Nefh	Tac1	Mrgprd	Th
Pvalb	Ntrk1	P2rx3	
	Calca		

Figure S9: Marker gene expression levels across 11 neuronal clusters identified using the *pcaReduce* algorithm.

References

[1] Chris Fraley and Adrian E Raftery. Mclust version 3: an r package for normal mixture modelling and model-based clustering. Technical report, DTIC Document, 2006.

[2] John A Hartigan and Manchek A Wong. Algorithm as 136: A k-means clustering algorithm. *Applied statistics*, pages 100–108, 1979.

[3] Vladimir Yu Kiselev, Kristina Kirschner, Michael T Schaub, Tallulah Andrews, Tamir Chandra, Kedar N Natarajan, Wolf Reik, Mauricio Barahona, Anthony R Green, and Martin Hemberg. Sc3-consensus clustering of single-cell rna-seq data. *bioRxiv*, page 036558, 2016.

[4] Yang Liao, Gordon K Smyth, and Wei Shi. The subread aligner: fast, accurate and scalable read mapping by seed-and-vote. *Nucleic acids research*, 41(10):e108–e108, 2013.

[5] Alex A Pollen, Tomasz J Nowakowski, Joe Shuga, Xiaohui Wang, Anne A Leyrat, Jan H Lui, Nianzhen Li, Lukasz Szpankowski, Brian Fowler, Peilin Chen, et al. Low-coverage single-cell mrna sequencing reveals cellular heterogeneity and activated signaling pathways in developing cerebral cortex. *Nature biotechnology*, 2014.

[6] Mark D Robinson, Davis J McCarthy, and Gordon K Smyth. edger: a bioconductor package for differential expression analysis of digital gene expression data. *Bioinformatics*, 26(1):139–140, 2010.

[7] Cole Trapnell, Lior Pachter, and Steven L Salzberg. Tophat: discovering splice junctions with rna-seq. *Bioinformatics*, 25(9):1105–1111, 2009.

[8] Dmitry Usoskin, Alessandro Furlan, Saiful Islam, Hind Abdo, Peter Lönnerberg, Daohua Lou, Jens Hjerling-Leffler, Jesper Haeggström, Olga Kharchenko, Peter V Kharchenko, et al. Unbiased classification of sensory neurone types by large-scale single-cell rna sequencing. *Nature neuroscience*, 18(1):145–153, 2015.

[9] Laurens van der Maaten. Barnes-hut-sne. *arXiv preprint arXiv:1301.3342*, 2013.

[10] Laurens Van der Maaten and Geoffrey Hinton. Visualizing data using t-sne. *Journal of Machine Learning Research*, 9(2579-2605):85, 2008.

[11] Chen Xu and Zhengchang Su. Identification of cell types from single-cell transcriptomes using a novel clustering method. *Bioinformatics*, page btv088, 2015.
